# Clinical relevance of a degree of extracapsular extension in a sentinel lymph node in breast cancer patients: a single-centre study

**DOI:** 10.1038/s41598-021-88351-z

**Published:** 2021-04-26

**Authors:** Tomasz Nowikiewicz, Andrzej Kurylcio, Iwona Głowacka-Mrotek, Maria Szymankiewicz, Magdalena Nowikiewicz, Wojciech Zegarski

**Affiliations:** 1 Department of Surgical Oncology, Nicolaus Copernicus University Ludwik Rydygier’s Collegium Medicum, Prof I. Romanowskiej 2, 85-796 Bydgoszcz, Poland; 2 Department of Clinical Breast Cancer and Reconstructive Surgery, Oncology Centre, Prof I. Romanowskiej 2, 85-796 Bydgoszcz, Poland; 3 Department of Surgical Oncology, Medical University, Lublin, Poland; 4 Department of Rehabilitation, Nicolaus Copernicus University Ludwik Rydygier’s Collegium Medicum, M. Sklodowskiej-Curie 9, 85-001 Bydgoszcz, Poland; 5 Department of Microbiology, Oncology Centre, Prof I. Romanowskiej 2, 85-796 Bydgoszcz, Poland; 6 Department of Hepatobiliary and General Surgery, A. Jurasz University Hospital, M. Sklodowskiej-Curie 9, 85-001 Bydgoszcz, Poland

**Keywords:** Cancer, Biomarkers, Oncology

## Abstract

In some breast cancer (BC) patients, an examination of lymph nodes dissected during sentinel lymph node biopsy (SLNB) demonstrates a presence of metastatic lesions and extracapsular extension (ECE) in a SLN. This study aimed to evaluate clinical relevance of ECE in BC patients. This is a retrospective analysis of 891 patients with cancer metastases to SLN, referred to supplementary axillary lymph node dissection (ALND), hospitalized between Jan 2007 and Dec 2017. Clinical and epidemiological data was evaluated. Long-term treatment outcomes were analysed. In 433 (48.6%) patients, cancer metastases were limited to the SLN (group I), in 61 (6.8%) patients the SLN capsule was exceeded focally (≤ 1 mm—group II). In 397 (44.6%) patients, a more extensive ECE was found (> 1 mm—group III). Metastases to non-sentinel lymph nodes (nSLNs) were diagnosed in 27.0% patients from group I, 44.3% patients from group II and in 49.6% patients from group III. No statistically significant differences were observed in long-term treatment outcomes for compared groups. The presence of ECE is accompanied by a higher stage of metastatic lesions in the lymphatic system. The differences in this respect were statistically significant, when compared to the group of ECE(−) patients. ECE, regardless of its extent, did not impact the long-term treatment results. ECE remains an indication for supplementary ALND and for other equivalent cancer treatment procedures, regardless of ECE size.

## Introduction

A diagnosis of an invasive form of breast cancer (BC) requires a sentinel lymph node biopsy (SLNB). In some patients, a pathological examination of dissected lymph nodes during SLNB reveals a presence of metastatic lesions (SLN+). The current standard for treatment of SLN(+) BC patients allows to spare lymph nodes without a need to perform an additional axillary lymph node dissection (ALND) in selected patients^1–4^. An extracapsular extension (ECE) is defined as a presence of lymph node capsule perforation by metastatic lesions with accompanying infiltration of perinodal space^5^. When ECE is found in SLN, an ALND appears to be the method of choice.

The Z0011 study initiated an era of conservative treatment in selected SLN(+) patients. The study also included cases with limited, however not specified ECE of metastatic SLN^6,7^.

An extracapsular extension is present in 22–55% of metastatic SLN^5,7–11^ and in 21.7–57.5% of nSLNs^5,7,10–13^. ECE is an important prognostic factor with negative effect on the overall survival (OS), and recurrence-free survival (RFS)^5,7,8,10,13^.

The extracapsular extension classification proposed by Katz et al. is based on two levels of lesion severity^14^. ECE exceeding 2 mm increases risk for a local and a regional recurrence of the disease by 27% and 33% respectively. Consequently, ECE not exceeding 2 mm presents with significantly lower risk of local and regional recurrence—18% and 22% respectively.

The aim of this study was to evaluate the clinical relevance of ECE in SLN in BC patients who underwent SLNB.

## Materials and methods

### Study details

**Figure 1:**
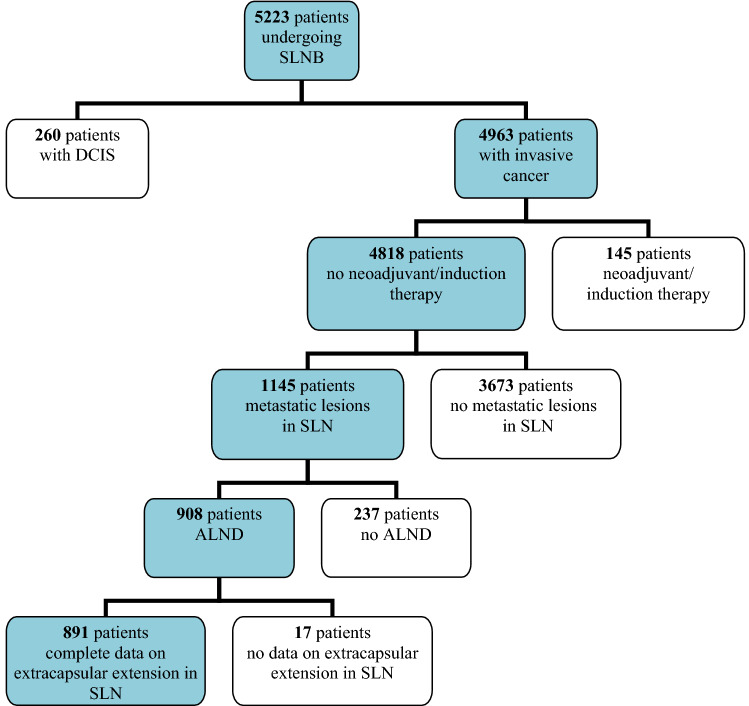
Patients’ qualification scheme.

Between January 2007 and December 2017 a total of 5223 BC patients underwent SLNB at The Department of Clinical Breast Cancer and Reconstructive Surgery, Oncology Centre—prof. Franciszek Łukaszczyk Memorial Hospital in Bydgoszcz. The retrospective analysis covers a group of 1145 SLN(+) patients. The need for informed consent from patients was waived by the Ethics Committee at the Nicolaus Copernicus University in Toruń, Collegium Medicum in Bydgoszcz (KB 675/2018 of Oct 30, 2018). 908 of them underwent completion ALND), 891 cases presented ECE in SLN. The analysis did not include 237 SLN(+) patients receiving conservative treatment without ALND. The remaining exclusion criteria included: pre-invasive BC, bilateral BC, neoadjuvant treatment or previously diagnosed different type of cancer. The design of the study is shown in Fig. 1.

Surgery and adjuvant treatment: radiation or systemic (chemo-, immuno-, or hormonal therapy) were performed according to current recommendations for BC treatment^1–4^.

### Evaluation of clinical data

Statistical analysis covered selected clinical and pathological data: primary tumour size, grading, histological and biological type of cancer, type and number of metastases to SLN and non-sentinel lymph nodes, the presence and size of ECE.

A three-level scale was used to describe the ECE presence and size. Three groups of patients were established: group I: no extracapsular extension in SLN; group II: focal extracapsular extension in SLN, up to 1 mm; group III: extensive extracapsular extension in SLN, above 1 mm (Table 1).

**Table 1:** Clinical characteristics of patients qualified for the study—univariate analysis.

Clinical data analysed	Group I No extracapsular extension in SLN n = 433 (%) [a]	Group II Focal extracapsular extension in SLN n = 61 (%) [b]	Group III Extensive extracapsular extension in SLN n = 397 (%) [c]	Group II + III Extracapsular extension in SLN n = 458 (%) [b + c]	[a] vs [b + c]	[b] vs [c]
**Histological form of invasive cancer**					0.0554	0.0209
NST	378 (87.3%)	48 (78.7%)	334 (84.1%)	382 (83.4%)	0.101	0.2891
Lobular	42 (9.7%)	9 (14.8%)	58 (14.6%)	67 (14.6%)	0.0251	0.9761
Other form	13 (3.0%)	4 (6.6%)	5 (1.3%)	9 (2.0%)	0.3173	0.0054
**Histological malignancy grade**					0.4081	0.4288
G1	12 (2.8%)	1 (1.6%)	11 (2.8%)	12 (2.6%)	0.8887	0.6031
G2	323 (74.6%)	51 (83.6%)	302 (76.1%)	353 (77.1%)	0.3898	0.1936
G3	89 (20.6%)	6 (9.8%)	76 (19.1%)	82 (17.9%)	0.3173	0.0767
No data	9 (2.1%)	3 (4.9%)	8 (2.0%)	11 (2.4%)	0.7414	0.1676
**Tumour size—pathological evaluation**					0.3497	0.8033
pT1	202 (46.7%)	24 (39.3%)	166 (41.8%)	190 (41.5%)	0.1211	0.7188
pT2	218 (50.3%)	36 (59.1%)	219 (55.2%)	255 (55.7%)	0.1118	0.5755
pT3	10 (2.3%)	1 (1.6%)	7 (1.8%)	8 (1.7%)	0.5485	0.9442
pT4	3 (0.7%)	0 (0.0%)	5 (1.3%)	5 (1.1%)	0.5287	0.3789
Tumour size—pathological evaluation	23.4 ± 11.1	24.0 ± 10.8	24.6 ± 10.8	24.5 ± 10.8	0.1244	0.7048
**Metastatic lesion type in SLN**					< 0.0001	0.4207
Macrometastases	376 (86.8%)	61 (100%)	386 (97.2%)	447 (97.6%)	< 0.0001	0.1868
Micormetastases	55 (12.7%)	0 (0.0%)	11 (2.8%)	11 (2.4%)	< 0.0001	0.1868
ITC	2 (0.5%)	0 (0.0%)	0 (0.0%)	0 (0.0%)	0.1443	1
Metastatic lesions in non-sentinel lymph nodes	117 (27.0%)	27 (44.3%)	197 (49.6%)	224 (48.9%)	< 0.0001	0.4407
**Metastatic lesions—pathological evaluation**					< 0.0001	0.2445
pN1	357 (82.5%)	44 (72.1%)	251 (63.3%)	295 (64.4%)	< 0.0001	0.177
pN2	68 (15.7%)	9 (14.8%)	97 (24.4%)	106 (23.1%)	0.0051	0.0949
pN3	8 (1.8%)	8 (13.1%)	49 (12.3%)	57 (12.5%)	< 0.0001	0.865
**The number of lymph nodes with metastatic lesions**					< 0.0001	0.5562
1	240 (55.4%)	21 (34.4%)	127 (32.0%)	148 (32.3%)	< 0.0001	0.7039
2	83 (19.2%)	15 (24.6%)	85 (21.4%)	100 (21.8%)	0.3271	0.5755
3	34 (7.9%)	8 (13.1%)	39 (9.8%)	47 (10.3%)	0.2113	0.4295
4 and more	76 (17.6%)	17 (27.9%)	146 (36.8%)	163 (35.6%)	< 0.0001	0.177
**Biological cancer type**					0.7852	0.2519
Luminal A	164 (37.9%)	29 (47.6%)	181 (45.6%)	210 (45.9%)	0.016	0.7795
Luminal B HER2-negative	140 (32.3%)	23 (37.7%)	112 (28.2%)	135 (29.5%)	0.3576	0.131
Luminal B HER2-positive	47 (10.9%)	4 (6.6%)	37 (9.3%)	41 (8.9%)	0.3421	0.4839
HER2-positive	23 (5.3%)	3 (4.9%)	15 (3.8%)	18 (3.9%)	0.3271	0.6672
Triple negative	33 (7.6%)	1 (1.6%)	28 (7.1%)	29 (6.3%)	0.4473	0.1052
No data	26 (6.0%)	1 (1.6%)	24 (6.0%)	25 (5.5%)	0.7263	0.1585
Multifocal tumour	101 (23.3%)	15 (22.2%)	88 (24.6%)	103 (22.5%)	0.7664	0.6729
Invasion of lymph vessels	41 (9.5%)	8 (13.1%)	49 (12.3%)	57 (12.5%)	0.1558	0.8649
Adjuvant CHTH	311 (71.8%)	44 (72.1%)	313 (78.8%)	357 (77.9%)	0.0258	0.2211
Adjuvant RTH	301 (69.5%)	57 (93.4%)	376 (94.7%)	433 (94.5%)	< 0.0001	0.7521
Mastectomy	209 (48.3%)	27 (44.3%)	185 (46.6%)	212 (46.3%)	0.7123	0.7293
BCT	224 (51.7%)	34 (55.7%)	212 (53.4%)	246 (53.7%)	0.7861	0.7955

The values of the analysed variables were compared between the ECE-free group of patients—ECE(−), and patients with ECE—ECE(+). The ECE size (group II vs group III) were additionally evaluated.

### Surgical techniques

All patients underwent a complete surgical treatment at single institution. SLNBs, ALNDs were performed by experienced BC surgeons in the same way during the study.

The isotope method, using ^99m^Tc with 75–100 MBq, administered on an albumin carrier (Nanocol) was used to identify the SLN. At the beginning of the study, a combined method (isotope and staining technique, with 1 ml of 2.5% Patent-V blue-dye added) was used to localize SLN. 34 of analysed patients underwent SLNB with dual technique.

The SLN was identified as a lymph node with the highest level of radiation (and/or containing the dye). In accordance with so called “Rule of 10%”, the LNs emitting radiation at a level exceeding 10% of the radio tracer emission value obtained for the SLN, were additionally resected^15^, along with every intraoperatively palpated and clinically suspicious LN.

The complete ALND (levels I–III) was performed in 891 patients.

### Statistical analysis

Statistical analyses were conducted using Statistica (the data analysis software system, TIBCO Software Inc., www.statistica.io, version 13.3). Relationships between categorical variables were assessed using the Chi-square test. The Student’s t-test or the Mann–Whitney’s U-test were used to compare continuous variables, depending on parameters of a distribution of a variable between groups.

The overall survival (OS) was defined as a time between the surgery (SLNB) date and patient’s death (regardless of its cause). The recurrence-free survival (RFS) was defined as a time between SLNB date and diagnosis of the first recurrence (or patient’s death, regardless of its cause). The overall survival time and the recurrence-free survival time were assessed using the Kaplan–Meier model, and compared using the log-rank test. In all statistical analyses, the cut-off value for the probability coefficient was set at p value of ≤ 0.05.

Sensitivity, specificity, positive predictive value (PPV) and negative predictive value (NPV) in predicting ECE in nSLNs(+) was determined.

The last follow-up visit was conducted in June 2019.

### Ethical approval

All procedures performed in studies involving human participants were in accordance with the ethical standards of the institutional and/or national research committee and with the 1964 Helsinki declaration and its later amendments or comparable ethical standards. This article does not contain any studies with animals performer by any of the authors. The study was approved by the Ethics Committee at the Nicholas Copernicus University in Toruń, Collegium Medicum in Bydgoszcz (KB 675/2018 of Oct 30, 2018).

## Results

In 433 (48.6%) patients, cancer metastases were limited to the SLN (group I), while in 61 (6.8%) patients the SLN capsule exceeded focally (group II). In the remaining 397 (44.6%) patients, a more extensive infiltration to the perinodal area was confirmed (group III). The average patient age was 57.0 years (ranging from 23 to 89 years).

The most common type of metastatic lesions in SLN in the ECE(−) group were macrometastases (86.6%), followed by micrometastases (12.7%). In the ECE(+) group, macrometastases and micrometastases were found in 97.6% and 2.4% of patients, respectively (p < 0.0001). Metastatic lesions to nSLNs were diagnosed in 27.0% of patients from group I and in 48.9% of the patients with ECE (p < 0.0001). Similar differences also concerned the pathological evaluation of LNs (pN; p < 0.0001), as well as a number of metastatically changed LNs (also p < 0.0001—Table 1).

Lobular BC was diagnosed in 9.7% of ECE(−) patients and in 14.6% of ECE(+) patients (p = 0.0251). Luminal A BC was diagnosed in 37.9% of ECE(−) patients and in 45.9% of ECE(+) patients (p = 0.016). Detailed data on clinical and pathological outcomes of patients included in the study is shown in Table 1.

### ECE and a risk of metastatic lesions in non-sentinel lymph nodes

ECE was found in 42.6% (234/550) of the nSLNs(−) patients and in 63.7% (227/341) of the nSLNs(+) patients (p < 0.0001).

The focal extracapsular extension in SLN was reported in 6.2% (34/550) of the nSLNs(−) and in 7.9% (27/341) of the nSLNs(+) patients (p = 0.3175), while extensive ECE was observed in 36.4% (200/550) and 57.8% (197/341) of nSLNs(−) and nSLNs(+) patients, respectively (p < 0.0001).

Sensitivity, Specificity, positive predictive PPV and NPV in predicting ECE in nSLNs(+) patients were 65.7%, 57.5%, 48.9%, 73%, respectively.

### Long-term treatment results

The median time for patient follow-up was 67 months (18–152 months). A recurrence of underlying disease was diagnosed in 96 (10.8%) patients. Among them, the local, regional, and distant cancer recurrence was found in 21.9%, 4.2% and 79.2% of patients, respectively.

**Figure 2:**
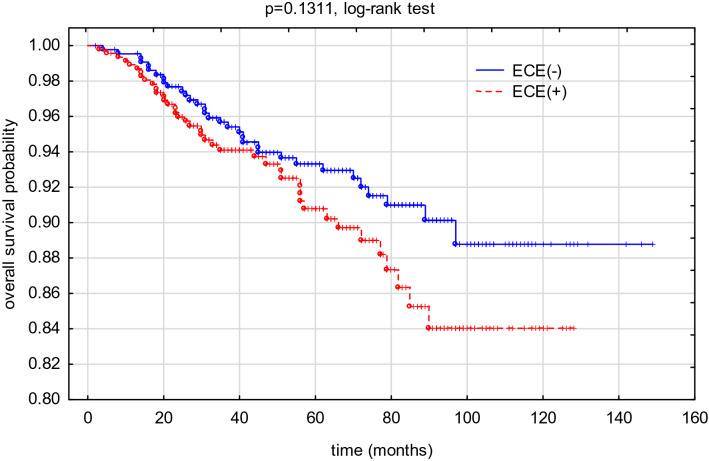
An overall survival probability (pOS) rate in the analyzed group depending on the presence of ECE.

**Figure 3:**
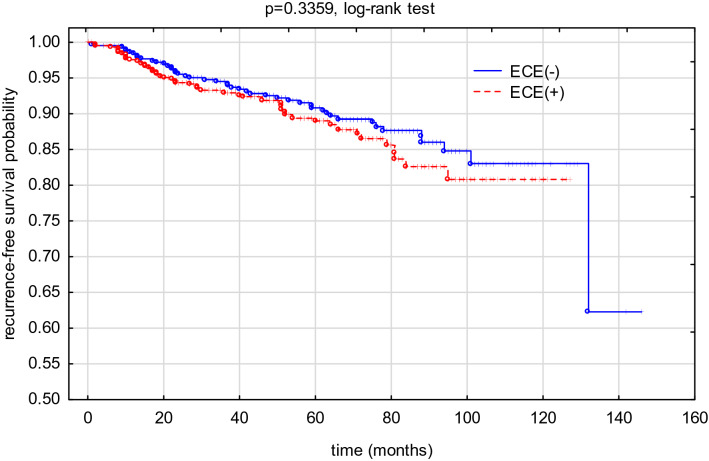
A recurrence-free survival probability (pRFS) rate in the analyzed group depending on the presence of ECE.

In total, 74 deaths were reported. Detailed data on long-treatment outcome is presented in Table 2. The probability of the 2- and 5-year overall survival (pOS), calculated for the study group, was 0.9689(± 0.0059) and 0.9212(± 0.0100), respectively. The probability of the 2- and 5-year relapse-free survival (pRFS), calculated for study group was 0.9502 ± 0.0074 and 0.9005 ± 0.0112, respectively. Detailed pOS and pRFS results are shown in Figs. 2, 3.

**Table 2:** Long-term treatment results.

Analysed clinical data	Group I No SLN extracapsular extension n = 433 (%) [a]	Group II Focal extracapsular extension in SLN n = 61 (%) [b]	Group III Extensive extracapsular extension in SLN n = 397 (%) [c]	Group II + III Extracapsular extension in SLN n = 458 (%) [b + c]	[a] vs [b + c]	[b] vs [c]
**Disease recurrence**	47 (10.9%)	5 (8.2%)	44 (11.1%)	49 (10.7%)	0.9402	0.4971
Local	13 (27.7%)	2 (40.0%)	6 (13.6%)	8 (16.3%)	0.2169	0.3265
Regional	3 (6.4%)	1 (20.0%)	0	1 (2.0%)	0.2896	nd
Distant metastases	35 (74.5%)	2 (40.0%)	39 (88.6%)	41 (83.7%)	0.5309	0.0955
Patient’s death	33 (7.6%)	2 (3.3%)	39 (9.8%)	41 (9.0%)	0.4719	0.1745

## Discussion

This study evaluated clinical relevance of ECE in BC metastases to SLN. Among available reports, our study analysed the largest number of cases from a single institution. The adopted method for data collection ensured an optimum consistency of the analysed clinical material.

As demonstrated previously, the ECE was the most important variable increasing risk of the metastatic lesions in nSLNs^9^. When compared to nSLNs(−) patients, infiltration to SLN perinodal tissues in the nSLNs(+) group was found in 71.4% patients when compared to the entire cohort. ECE proved to be even more clinically significant than the character (micro- or macrometastases) of metastatic lesions in SLN. Consequently, ECE allowed development of statistical tools for prediction of metastatic lesions in nSLNs(+) patients^9,16,17^. Our study indicates that ECE statistically increase percentage of nSLNs(+) cases when compared to ECE(−) patients, which is in accordance with previous reports^5,7,10–12,18^. Nonetheless, we did not find any significant influence of the ECE size on other variables related to the stage of metastatic lesions in the lymphatic system. It should be emphasised that the pN stage and number of metastatic LNs differed significantly between ECE(−) and ECE(+) groups.

Similar results were presented by Schwentner et al., where 343 SLN(+) patients, including 104 cases of ECE(+) were analysed^8^. Of the variables compared by the authors, significant differences concerned the pN stage, type of metastatic lesions in SLN, and the size of primary tumour.

According to the results of the studies conducted by Gooch et al.^12^, ECE is more frequent in patients with primary multifocal lesions, invasion of lymph vessels, presence of steroid hormone receptors and large tumours.

Our study did not demonstrate the influence of the ECE on long-term treatment results. Nevertheless, patients with ECE were more frequently qualified for adjuvant CHTH and adjuvant RTH when compared with ECE(−) patients (77.9% vs 71.8% and 94.5% vs 69.5%, respectively). Noteworthy, the differences in RTH administration were not influenced by the surgical treatment, since BCT rate was comparable in both analysed groups. This could therefore influence the achieved treatment results.

In a study conducted by Schwentner et al., different treatment results were achieved. The ECE presence was associated with worse 5-year OS, but it had no influence on 5-year DFS^8^. Similar results were also presented by Drinka et al.^5^ and Shigematsu et al.^7^. However, in SLN(+) patients, ECE is considered as an independent DFS prognostic factor^7,18,19^.

Similar observations concern the extent of ECE. ECE below 2 mm has no influence on the recurrence rate, and is similar to ECE(−) patients^11,18,20^. However, due to retrospective nature of the studies, lack of the complete evaluation of the axillary treatment on long-term outcomes and relatively small patient groups, these results should be interpreted with caution.

Despite gradual limiting of indications for surgical treatment in SLN(+) patients^1,6–8,21–23^, unambiguous evidence for management of patients with ECE is not available. However, since significant differences in long-term treatment outcomes in ECE(+) patients were shown, further prospective studies are warranted.

This study contains certain limitations. Due to the three-level method for ECE size evaluation (no ECE, ECE up to 1 mm, ECE exceeding 1 mm), as well as a retrospective character of the study, only three groups of patients could be distinguished. This significantly limited the scope of statistical calculations, including determination of other groups of patients.

## Conclusions

Although with no effect on long term treatment outcomes, ECE in SLN + BC patients is accompanied by higher pN stage and therefore seems to be an justified indication for ALND and complementary adjuvant treatment.

## Data Availability

This study is a retrospective analysis based on clinical data from the hospital cancer registry. Therefore, informed consent from patients were not required for this study. Data from the hospital cancer registry provided anonymised patient data.
